# Field vaccination of locally-owned cattle against malignant catarrhal fever under environmentally challenging conditions in Tanzania

**DOI:** 10.1016/j.vaccine.2024.126587

**Published:** 2025-01-25

**Authors:** Samuel Bainbridge, Tauta Mappi, Sarah Cleaveland, Choby Chubwa, Alicia Davis, Dawn Grant, Tito Kibona, Shedrack Bwatota, Freja Larsen, Samson Lyimo, Fadhili Mshana, Ann Percival, Gabriel Shirima, Bakari Mtili, Felix Jackson Musyangi, Rigobert Tarimo, Felix Lankester, George Russell

**Affiliations:** a School of Biodiversity, One Health and Veterinary Medicine, College of Medical, Veterinary and Life Sciences, University of Glasgow, Glasgow, United Kingdom; b Nelson Mandela African Institution of Science and Technology, Tanzania; c Ministry of Livestock and Fisheries, Tanzania; d School of Social & Political Sciences, University of Glasgow, Glasgow, United Kingdom; e Moredun Research Institute, Pentlands Science Park, Midlothian, United Kingdom; f Paul G. Allen School for Global Health, Washington State University, Pullman, USA; g Global Animal Health Tanzania, Arusha, Tanzania

**Keywords:** Malignant catarrhal fever, Vaccine, Immunogenicity, Safety, Pastoral, Cattle

## Abstract

Malignant catarrhal fever (MCF), caused by alcelaphine herpesvirus-1 (AIHV-1) transmitted from wildebeest, is a lethal cattle disease with significant impacts on East African pastoralists. Development of a live attenuated MCF vaccine has prompted research into its use in communities at risk. This study reports results from the first utilisation of the MCF vaccine in locally-owned cattle under field conditions. The study involved a primary two-dose course vaccination of 1634 cattle, followed a year later, by boost vaccination of 385 of these cattle. It aimed to: (a) evaluate the antibody response to a two-dose AlHV-1 primary vaccination course, including initial response, antibody levels after one year, and clinical events post-vaccination; (b) assess how factors like age, reproductive status, body condition, and breed influence the initial response; and (c) compare antibody responses to single- and two-dose booster protocols one year after primary vaccination. Analyses were carried out using linear mixed-effects models and paired *t*-tests.

Clinical incidents were reported in 11/1634 cattle vaccinated during the primary course and in 0/385 cattle during the boost regimens. The primary vaccination resulted in a 9-fold increase in comparison to pre-vaccination antibody levels and the response was consistent across animals of different ages, reproductive statuses and body conditions. While antibody levels declined 11 months after primary vaccination, they remained high, and a single-dose booster vaccination was sufficient to elicit a strong immune response, with only marginal increases after a second booster.

The study provides evidence of high immunogenicity and low incidences of clinical events of the vaccine in cattle across individual host factors and immunologically vulnerable groups, under prevailing environmental conditions. It also indicates the utility of a single-dose booster regimen. These findings will support progress towards commercial production and larger-scale adoption which could generate important benefits for the livelihoods, and sustainability of pastoral livestock systems.

## Highlights

• Zebu cattle were vaccinated against malignant catarrhal fever using an attenuated live virus in remote northern Tanzania.
• Strong antibody responses followed a two-dose vaccination and single booster, unaffected by age, reproductive status, or condition.
• No major adverse effects were reported following primary or boost-vaccination.

## Graphical abstract

**Figure:**
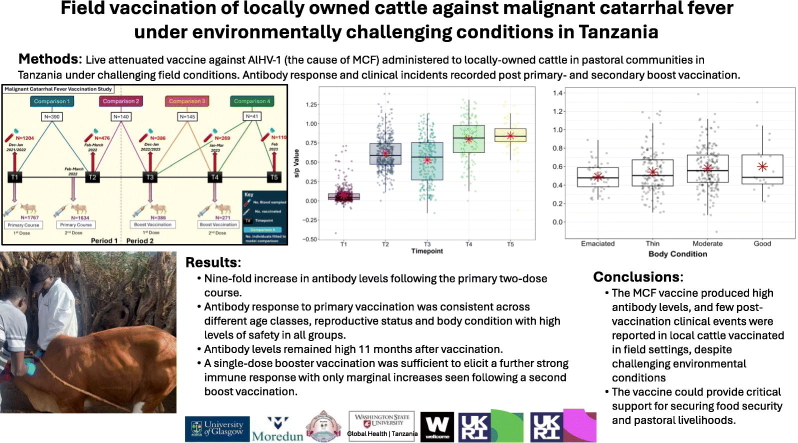

## 1 Introduction

Malignant catarrhal fever (MCF) is a fatal disease of cattle, caused by certain herpesviruses of genus *Macavirus* subfamily *Gammaherpesvirinae*. The disease is characterised by efficient spread of MCF viruses and lifelong infection in their respective natural hosts, without obvious clinical signs, while infection of MCF-susceptible host species leads to MCF [1]. The disease is typified by proliferation of activated infected CD8 T cells, infiltrating multiple tissues and causing systemic inflammatory and necrotic lesions that usually lead to death within days or weeks of the initial clinical signs [[1], [2], [3]]. The major forms of MCF worldwide are sheep-associated MCF, caused by ovine herpesvirus 2 and seen wherever sheep are raised close to susceptible species such as cattle, deer and bison; and wildebeest-associated MCF (WA-MCF), caused by alcelaphine herpesvirus-1 (AlHV-1) that is transmitted from wildebeest across eastern and southern Africa [1]. The disease poses a substantial burden on the livelihoods and food security of pastoralists in East Africa and is a principal factor driving land-use conflict at the borders of wildlife-protected areas 4. Across affected communities, MCF is perceived to be among the most important cattle diseases with reported losses from fatal disease ranging from 3 % to 12 % per year [[4], [5], [6]].

Further impacts arise from the need to avoid MCF, with pastoralists adopting strategies that involve moving cattle away from high-quality grazing lands used by wildebeest during the calving season, to less productive pastures that are often far away from the permanent household [[4], [5], [6]]. In many East African ecosystems, wildebeest move outside the borders of wildlife protected areas during the calving season to access mineral-rich pastures needed for lactation. However, access to these high-quality pastures at the end of the dry season (when wildebeest start to calve) is also critical for cattle to regain body condition and for conception. A further problem is that by moving cattle to temporary pastures for several months every year, family members remaining at the permanent household (mostly women and children) have much less access to milk [7], which is a critical component of pastoralists' diet and essential for children, improving nutritional health, cognitive development, and educational attainment [[8], [9], [10]].

The development of an attenuated vaccine against AlHV-1 creates important opportunities for pastoralists to protect cattle against MCF deaths and to use grazing lands that have previously been inaccessible [7]. Pastoralists are currently facing intense pressures on grazing land because of climate change which is manifesting in East Africa as more frequent and prolonged droughts [11,12]; the widespread conversion of rangeland to crop-based agriculture [13,14]; and an expansion of exclusionary land use policies for wildlife conservation and wildlife-based tourism [15,16]. In the Serengeti ecosystem in Tanzania, one of the key MCF hotspots, these challenges have been compounded by the rise in wildebeest numbers (increasing more than 6-fold since the 1960s) [17] and the increasing utilisation by wildebeest of mixed land-use grazing areas outside the Serengeti National Park [14]. From a conservation perspective, the vaccine could support the viability of mixed livestock-wildlife land-use systems that are the only areas in East Africa, outside of protected areas, that still sustain abundant populations of large wildlife [[18], [19], [20]].

Despite a long history of unsuccessful attempts at developing an inactivated AlHV-1 virus vaccine for cattle [21], considerable progress has now been made with the development of a tissue-culture attenuated AlHV-1 virus. This vaccine, derived by serial culture of the C500 viral isolate from a Kenyan MCF case, successfully protected Holstein/Friesian cattle from experimental transmission under laboratory conditions [22]. Subsequent field trials, under conditions of natural challenge (exposure of cattle to wildebeest calves), showed that the vaccine provided 80 % protection against fatal infection in Boran and Boran/Friesian cattle in Kenya [23], and 56 % protection against non-fatal infection in a study involving shorthorn zebu cattle in Tanzania [24].

Experimental challenge studies indicate that, while a two-dose primary course provided good protection for up to six months, immunity waned in subsequent months, in parallel with the antibody responses [25], so annual vaccination may be needed to ensure protection. For annual vaccinations to be a feasible option, protection from a single-dose booster course of vaccination would offer considerable advantages, particularly when considering vaccination in remote and hard-to-reach pastoral communities [15]. A further consideration is in the timing of vaccination, as the accessibility of herds is particularly challenging at the end of the dry season, immediately prior to the MCF risk season. The longer the duration of immunity, the greater the flexibility in timing, allowing vaccination to be carried out at times of year when cattle are in better condition, less dispersed, and when vaccination could be linked with other routine veterinary activities.

While data from earlier studies have consistently demonstrated high levels of antibody following the two-dose primary immunisation protocols, cattle in these studies were managed as research herds, with supplemental feeding and herd health interventions, including anthelminthic treatment [23,24]. However, for locally owned and managed cattle the growing pressure on grazing lands and the increasing frequency of drought is likely to be intensifying the nutritional stresses facing cattle [26]. In this study, we therefore aimed to investigate vaccine immunogenicity, in terms of virus-specific antibody responses, when it is delivered to local-breed cattle that are owned and managed under prevailing environmental conditions.

This study presents results from a series of immunogenicity trials of AlHV-1 vaccination in locally-owned cattle carried out under field conditions. The study aimed to: (a) determine the response to a primary two-dose course of AlHV-1 vaccination delivered to locally-owned African shorthorn zebu cattle managed under local conditions, specifically evaluating the initial antibody response, antibody levels approximately one year after the primary course and clinical events observed following vaccination; (b) investigate the impact that various factors (e.g. age group, reproductive status, body condition score and breed) have on the initial antibody response to this primary course of AlHV-1 vaccine; (c) evaluate and compare antibody responses to single- and two-dose booster vaccination protocols approximately one year after primary vaccination. Analyses were carried out using linear mixed-effects models and paired *t*-tests.

## 2 Materials and methods

### 2.1 Study area and household selection

**Fig. 1:**
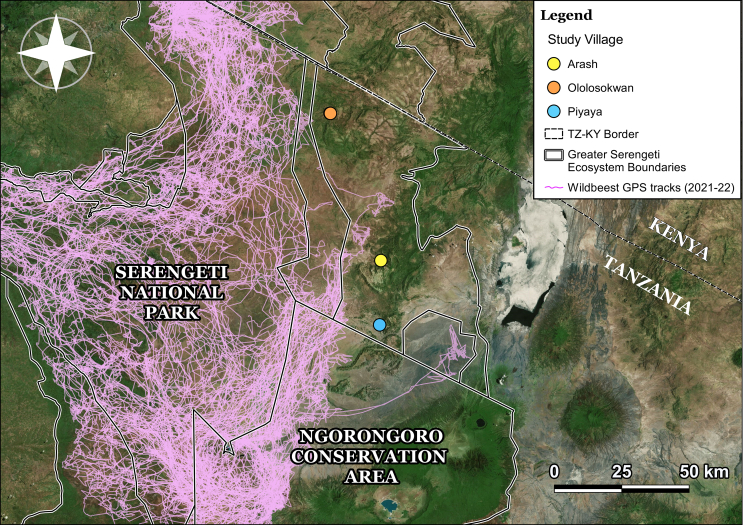
Map showing the main study villages (Arash, Piyaya and Ololosokwan) in Northern Ngorongoro district, Tanzania, where research focused on the effects of the Malignant Catarrhal Fever vaccination. GPS collar data illustrating wildebeest movements from 2021 to 2022 is shown on the map (Wildebeest data from Serengeti Biodiversity Project, Prof. Grant Hopcraft).

The study was conducted in three communities, Arash, Piyaya and Ololosokowan in the Ngorongoro District, Tanzania, known to be at risk from MCF [6] and where there are still substantial annual movements of wildebeest into grazing lands during the calving season (Fig. 1). Following initial meetings with district authorities and communities to introduce the study, households were selected at random from a list of households obtained from the village headquarters. Overall, 69 households were recruited to take part in this vaccination study, comprising 25 households from Arash, 19 from Piyaya and 25 from Ololosokwan.

Following selection of households, each was visited with detailed information provided to the head of the household about the purpose of the study, the risks involved (including explanation of an anticipated vaccine efficacy of 80 %) and written informed consent provided (see Supplementary Information 6.2). Each household was invited to bring a maximum of 30 cattle for vaccination, administered free of charge, but it was not known in advance how many cattle owners would choose to vaccinate. A unique household ID was assigned to each participant to ensure anonymity, and ear-tag numbers were assigned to individual cattle and used as reference points in the data collection process and vaccination record.

*Sample size calculation*. A sample size was determined based on detecting a difference in the mean antibody response (measured by the difference in ELISA s/*p* values, see below) of 0.05 between different groups of animals, including animals with different body condition scores, reproductive status and receiving different vaccination protocols (e.g. single- versus two-dose booster vaccination). Assuming a standard deviation of 0.15 (derived from baseline serology data [24]), with β = 0.8 and α = 0.05, 150 animals would be needed in each group to detect this size of effect. Given expectations of high owner demand for vaccine and allowing for loss to follow up, we anticipated that recruitment of 69 households would allow us to generate data from substantially more than the 600 cattle that would be needed to compare antibody response across four categories of body condition score which was considered the principal variable of interest in this study.

### 2.2 Primary vaccination and blood sampling (2021−2022)

Following the consenting procedures, arrangements were made to visit the recruited households early in the morning for vaccination and blood sampling. Animals identified by the farmer were brought forward for vaccination and manually restrained. An ear tag was fitted to each animal, the ear tag number recorded and up to 10 mls of blood was collected by jugular venipuncture into a plain vacutainer (Becton Dickinson) to obtain a baseline pre-vaccination blood sample (**T1**).

The age was recorded as reported by owners, but where the research team had reason to question this response, the age was checked and recorded from the animal's dentition [27]. The body condition score of each animal was recorded as emaciated, thin, moderate, or good, following standard methods based on visibility of ribs, spine, and spinous processes [28]. Consistency of scoring among enumerators was assessed prior to vaccination and periodically throughout the program.

The MCF vaccine virus (Batch Numbers 04/2021 and 08/2022) was produced by the Moredun Research Institute (UK) as described previously [25]. Briefly, attenuated AlHV-1 strain C500 was propagated in bovine turbinate cells and cell-free, sterile culture supernatant containing approximately 10^6^ TCID 50/ml was harvested and stored frozen until required. On the day before vaccination, sufficient vaccine was removed from the freezer and thawed overnight at 4 °C and transported in cool boxes or a mobile fridge to the household. Immediately prior to vaccination, 8 mls of adjuvant, Emulsigen® (20 % v/ v) (MVP, Omaha, USA), was added to each 32 ml tube of vaccine virus suspension and the tube inverted several times to ensure thorough mixing. Once reconstituted, vaccine was stored in cool boxes or the vehicle fridge and was used no longer than 36 h after the Emulsigen® was added. 1 ml of the reconstituted vaccine (0.8 ml of virus suspension and 0.2 ml of Emulsigen®) was inoculated intramuscularly in the neck using a 2 ml syringe and 20 g 1.5 in. needle. Following vaccination, the animal was kept under close observation for 30 min for detection and potential treatment of adverse reactions by the research team. Owners were asked to report any subsequent adverse clinical events to the government livestock field officer for further investigation and treatment if required (see Section 2.3).

All households were re-visited between 24 and 45 days after the first vaccination and a second dose of vaccine administered following the same vaccination protocol, as above (primary course, dose 2). During this visit, owners were also asked about any deaths or adverse clinical events in vaccinated animals. Households were re-visited between 24 and 35 days after the second dose of vaccine for collection of a post-vaccination blood sample (**T2**), following the protocol described above. No blood samples were collected after the first dose of vaccine given results from earlier studies indicating that no immune response has been detectable until after the second dose [24,29].

### 2.3 Investigation of adverse events following vaccination

Adverse events were recorded through: (i) direct observation of vaccinated cattle during the immediate (30-min) post-vaccination period; (ii) owner reports of adverse events conveyed to the research team by livestock field officers; and (iii) data collected during repeat vaccination and post-vaccination blood sampling visits. Where a field investigation was possible, a clinical examination and/or post-mortem was carried by the livestock field officer and/or veterinarians within the research team. Although the study was not designed to assess efficacy, cases of suspected MCF were investigated where possible and samples collected for real-time PCR diagnosis using protocols established at Nelson Mandela Institute of Science and Technology (NM-AIST) [30].

### 2.4 Boost vaccination and blood sampling (2022−2023)

A further round of vaccination and blood sampling (period 2, P2) was carried out in the following year (mid-December 2022 to mid-February 2023) to: (i) evaluate antibody levels approximately one year after the primary course, and (ii) evaluate and compare antibody responses to a single- and two-dose booster vaccination protocols.

**Fig. 2:**
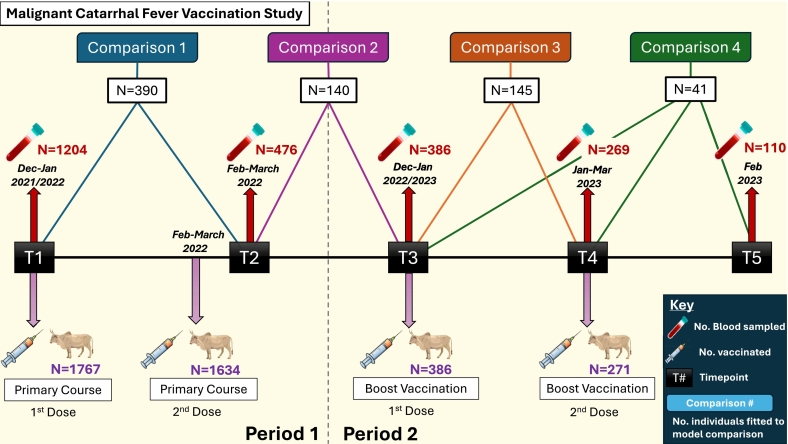
Scheme showing the timeline of vaccination and blood sampling for assessment of antibody responses following primary and boost vaccination. The numbers of animals shown for each comparison group includes on those for which data from all the variables included in the model were available.

Following informed consent of cattle owners involved in the initial period of the study, households were re-visited, and owners asked to bring forward all previously vaccinated cattle for a further round of vaccination. For this period of the study, a blood sample was collected prior to booster vaccination (**T3**) to assess the change in antibody levels over the 10–12-month interval since the primary course of vaccination. Households were then re-visited at 28–29 days after the first booster vaccination for a further blood sample and a second dose of vaccine (booster vaccination, dose 2) (**T4**), and again 28 days after the second booster dose for a post-vaccination blood sample (**T5**). The full timeline of vaccination and blood sampling for T1-T5 can be seen in Fig. 2. Protocols for vaccination, blood sampling, body condition score measurements and recording of adverse events followed those described above.

### 2.5 Serological assays

Serum was obtained by centrifuging coagulated blood at 3000 rpm for 10 min on the day of sampling. Serum was stored in the field and transported to the laboratory in a mobile freezer at approximately -20 °C, and subsequently stored at the laboratory at -80 °C. Serum samples were tested for WA-MCF antibodies at the NM-AIST using an indirect ELISA developed at Moredun Research Institute [25,31]. Briefly, pairs of adjacent rows of 96- well microtitre plates (Greiner, high protein binding) were coated with 50 μl of 5 μg/ml virus-positive or virus-negative antigen in 0.1 M carbonate buffer, pH 9.6. Individual samples of serum were then applied in duplicate to positive and negative antigen wells at a dilution optimised in previous experiments (1/500). A similar dilution of an AlHV-1-positive serum was included on each plate as a positive control and to ensure reproducibility between assays. A known negative serum sample was included with each test sample series. Antibody bound in each well was detected using 1:1000 dilution of rabbit anti-bovine IgG-Horseradish Peroxidase conjugate (Sigma). The plates were washed, and Sureblue TMB-peroxidase substrate (KPL) applied for five minutes. The reaction was stopped by the addition of 0.1 M HCl, and the plates were read at 450 nm in a plate reader. ELISA values (difference between means of positive and negative antigen wells for each sample) were normalised between plates by calculation of the sample-to-positive (s/p) ratio for each test sample, based on the positive control samples included on each plate, as described previously [32]_._ A cutoff of 0.06 for seropositivity in this assay was defined by analysis of 88 cattle samples from a herd with no previous record of MCF or contact with sheep [31].

### 2.6 Data analysis

Data analysis was conducted in R statistical software [33]. For each comparison, we used a complete case analysis, including only cattle with data available for all relevant variables and timepoints. This approach minimised concerns about non-random missing data.

To investigate the factors influencing the antibody response in cattle across different timepoints, we used linear mixed-effects models using the lmer() function from the lme4 package [34]. For the Comparison 1 model (Fig. 2.), we included individual-level factors that might affect the response to primary vaccination such as age, reproductive status, breed, village, and body condition, depending on the data available (study aims (a) and (b)). The response variable was the s/p difference between timepoints T2 and T1, with household ID included as a random effect. This approach was chosen due to limitations encountered during model fitting when using individual s/*p* values as the response variable and individual ID as a random effect. The s/p difference was log-transformed because this resulted in a better fit based on the distribution of the data and allowed the assumptions of the model to be met.

For Comparison 2 (T2-T3) and Comparison 3 (T3-T4), our aims were to assess the decline in antibody level approximately 1 year after primary vaccination (study aim (a)) and the increase after a single-booster (study aim (c)). To analyse these comparisons, we performed paired *t*-tests on antibody levels for each pair of timepoints.

For the Comparison 4 linear mixed-effects model, the only explanatory variable included was timepoint to account for individual cattle's s/*p* value at T3, T4 and T5 with individual ID fitted as a random effect to account for repeated measures (study aim (c)).

Although not a primary study aim, we investigated whether village was associated with pre-exposure status pre-vaccination. To do this we applied a logistic regression model using the generalised linear model (GLM) function in R. The binary outcome variable indicated whether an individual was classified as pre-exposed (1) or not (0). This was based on showing baseline s/*p* values >0.06, which is considered evidence of MCF seropositivity in UK cattle [31,32]. The model was fit using a binomial family, with a logit link function to model the log-odds of pre-exposure. Further information on the models fitted for each comparison is provided in the Supplementary Information (Section 6.1, Table 1), which shows a summary description for each analysis.

## 3 Results

### 3.1 Vaccination and blood sampling

Fig. 2 shows the number of cattle vaccinated at each time point along with the number from which blood samples were collected for serological analysis.

### 3.2 Clinical events

No adverse effects were recorded by the research team during the immediate post- vaccination observation period (30 min after vaccination) in any cattle vaccinated across the whole study.

During P1 (December 2021–March 2022), a total of 11 clinical events, unconnected to MCF, were reported by owners from the 1634 cattle vaccinated, of which six events involved a full recovery and five involved fatal incidents. Although this study was not designed to assess efficacy, cases of suspected MCF were investigated where possible and MCF was confirmed in two fully vaccinated animals (A-238 and A-250) several months after vaccination following exposure to wildebeest calves. Full details are provided in Supplementary Information (Section 6.3 and 6.4).

No clinical events were reported by owners or observed by the research team in any of the 385 cattle receiving booster vaccinations during the P2 period of the study.

### 3.3 Baseline seroprevalence

The mean s/*p* value for cattle at T1 prior to vaccination was 0.055 (s.d. ± 0.068), with 135/390 (34.6 %) showing baseline s/*p* values greater than 0.06 (Mean = 0.12, s.d. ± 0.061) and were therefore classified as seropositive [31,32].

The binomial GLM revealed a significant difference in the distribution of animals classified as seropositive across villages prior to vaccination. For Ololosokwan (24/49, 48.9 % seropositive), the estimate of the model was positive and significant (Estimate = 0.66, Std. Error = 0.32, z = 2.05, *p* = 0.04), indicating higher log odds of pre-exposure compared to Arash as the reference level (70/211, 33.2 % seropositive). In contrast, the estimate for Piyaya (41/130, 32.0 % seropositive) was not significant (Estimate = −0.075, Std. Error = 0.24, z = −0.31, *p* = 0.75), indicating no statistical difference in pre-exposure between Piyaya and Arash. Model results are reported in full in Supplementary Information (Section 6.5).

### 3.4 Antibody responses to vaccination

*Period 1: Response to primary two-dose vaccination.*

In P1, a total of 1634 cattle received the primary two-dose course of vaccination. Among them, 558 cattle were from Arash, 729 from Ololosokwan, and 347 from Piyaya. Of these, 390 individuals were sampled and serologically tested at both T1 and T2 and possessed data for all explanatory variables to be included in the Comparison 1 model. All animals except for one individual showed an increase in antibody levels when sampled at four weeks after the second dose of the primary course of vaccination i.e., the difference in antibody s/*p* value between T2 and T1 was greater than zero. At T2 the mean s/p value was 0.61 (s.d. ± 0.21), which indicated a greater than 9-fold average increase in the response in comparison to levels pre- vaccination.

**Fig. 3:**
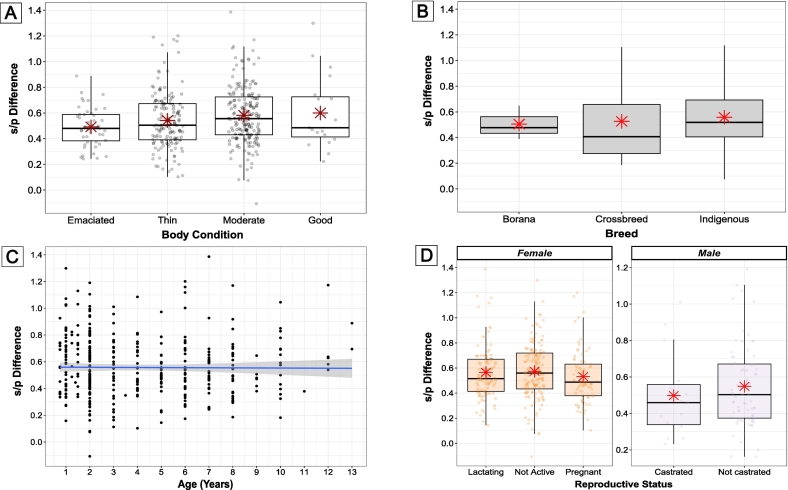
Figure illustrating various factors affecting s/p difference values between T1 and T2 in Comparison 1. (a) Box and whisker plot showing the relationship between s/p difference and body condition levels (‘Emaciated’, ‘Thin’, ‘Moderate’, and ‘Good’), with red asterisks indicating the mean for each condition. (b) Box and whisker plot comparing s/p difference across breeds (‘Borana’, ‘Crossbreeds’, and ‘Indigenous’), with red asterisks marking the mean for each breed. (c) Scatterplot showing the relationship between age and s/p difference, with a fitted regression line (y ∼ x) highlighting potential trends. (d) Box and whisker plot depicting s/p difference by reproductive status (‘Lactating’, ‘Not Active’, ‘Pregnant’ for females, and ‘Castrated’, ‘Not castrated’ for males), with red asterisks indicating the mean for each group. (For interpretation of the references to colour in this figure legend, the reader is referred to the web version of this article.)

**Fig. 4:**
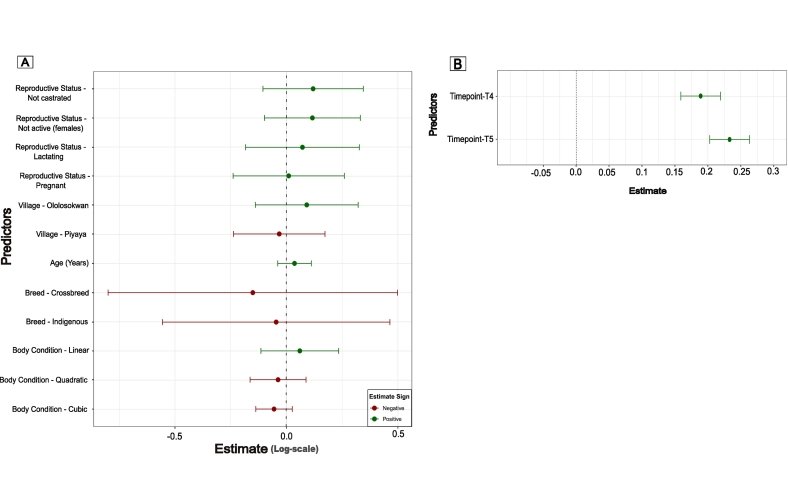
Coefficient plot summarising the results of linear mixed-effects models investigating factors influencing the antibody response in cattle across different timepoints. (a) Comparison 1, with individual-level factors on the y-axis, and the values of those parameter estimates based on a logarithmic scale, with log-transformed s/p difference between timepoints T2 and T1 as the response variable. (b) Comparison 4, the model focused on the effect of timepoint (T3, T4 and T5) included on the y-axis, on s/*p* values. The coefficient plot shows the estimated effects of each predictor variable along with 95 % confidence intervals.

The antibody responses following primary vaccination (difference between s/*p* values at T1 and T2) are shown in relation to body condition, breed, age (years), and reproductive status in Figs. 3a-3d. None of the explanatory variables included in the model had a statistically significant effect on the antibody response (*p* > 0.05) as shown in Fig. 4a (Table 4, Supplementary Information Section 6.7).

*Period 2: Assessment of decline in antibody levels.*

**Fig. 5:**
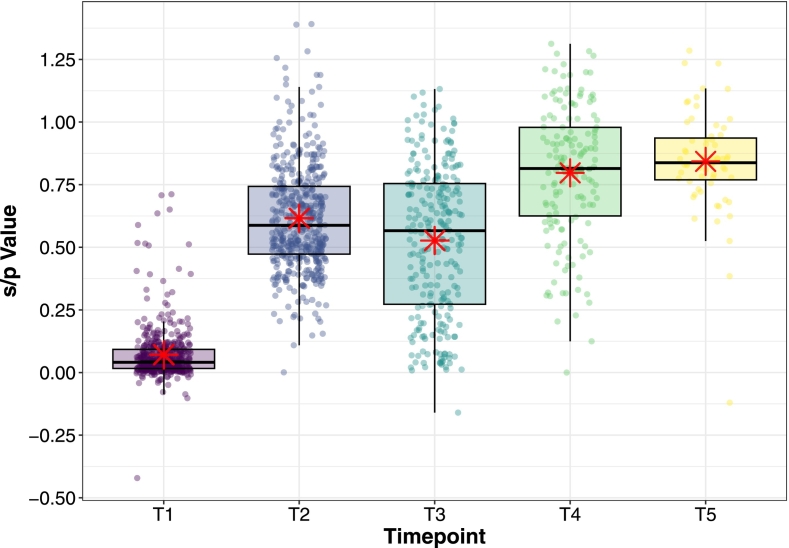
Box and whisker plot depicting s/p values for each timepoint included in the malignant catarrhal fever vaccination study (T1, T2, T3, T4 and T5) with red asterisks indicating the mean for each group. The sample used for this plot includes all animals for which a blood sample was available for the timepoint. (For interpretation of the references to colour in this figure legend, the reader is referred to the web version of this article.)

Over the 11 months following the primary two-dose vaccination there was a statistically significant decline (Mean = −0.11, *t* = 3.85, df = 139, *p* < 0.001) in ELISA s/*p* values detected by the paired *t*-test, from a mean of 0.60 (s.d. ± 0.22) at T2 to a mean s/p value of 0.49 (s.d. ± 0.30) at T3 (Comparison 2), but these values remained strongly positive in comparison to pre-vaccination levels at T1 (Fig. 5).

*Period 2: Comparison of antibody responses following single and two-dose boost vaccination.*

Antibody levels measured four weeks after the first dose of the booster vaccination protocol at T4 (0.80 ± s.d. 0.27) were significantly higher in the paired t-test (Mean = 0.32, *t* = 16.97, df = 145, *p*-value <2.2e^−16^) than pre-boost levels at T3 (0.48 ± s.d. 0.28) (Comparison 3, *N* = 145) and exceeded those recorded after the two-dose course of primary vaccination (0.61 ± s.d. 0.21).

In the linear mixed model investigating Comparison 4, the results showed there was a statistically significant effect of both T4 (single-dose booster) (Estimate = 0.19, Std. Error = 0. 015, z = 12.29, *p* < 0.001) and T5 (second-dose booster) (Estimate = 0.23, Std. Error = 0.015, z = 15.14, p < 0.001) on pre-boost levels at T3 shown in Fig. 4b. (Table 5, Supplementary Information Section 6.7).

There was only a small increase in overall mean values following the second booster dose at T5 (0.88 ± s.d. 0.16) which was weakly significant and had a minimal effect size of 0.04 (Fig. 4b).

## 4 Discussion

This study reports antibody responses and the incidence of clinical events associated with a new MCF vaccine following its use in in local cattle, mostly African shorthorn zebu, under field conditions in northern Tanzania. Building on previous randomized controlled field trials of vaccine safety and efficacy carried out in Kenya and Tanzania [23], [24] this study includes animals of different ages, reproductive statuses, and body conditions and investigates antibody responses following booster vaccination. The study demonstrated four key findings: (1) robust antibody responses were recorded in cattle given a two-dose primary course of vaccination regardless of age, reproductive status, breed or body condition; (2) antibody levels declined less than expected over the 11 months following primary vaccination; (3) a single-dose boost vaccination resulted in substantial increases in antibody level above that achieved following primary vaccination while a second booster dose gave only a small additional benefit; (4) very few clinical incidents were reported following primary or boost-vaccination, consistent with high levels of vaccine safety, and despite many animals being in very poor body condition. These results shed light on key aspects of the vaccine's performance and provide insights that can impact the optimisation of future vaccination strategies and contribute to the commercialisation of the vaccine for use in communities at risk of MCF.

Antibody responses to primary vaccination were robust, with all but one animal showing an increase in antibody levels and, overall, vaccination resulting in a greater than 9-fold increase in s/p compared to baseline values. This significant result demonstrates that the high antibody levels observed in research herds in previous studies can be successfully replicated in cattle under local management and conditions [23,24]. The vaccine also demonstrated consistently high immunogenicity irrespective of body condition, breed, age, reproductive status, and village of origin (Fig. 4a), all factors that can affect immunity and response to vaccination in cattle [35,36] and that require consideration in the design of vaccination programs [35,36]. To ensure maximum protection of cattle against MCF, the study was designed so that the primary vaccination course would be administered shortly before the wildebeest calving season when AlHV-1 transmission risk is known to be at its highest. However, in 2021/22, this coincided with the end of a severe drought creating extremely challenging conditions with cattle generally in very poor condition. Despite these conditions, the apparent robustness of immune responses of local cattle in this study to the MCF vaccine generates confidence in the effectiveness of MCF vaccination strategies, even in the face of extremely challenging environmental conditions that are likely to be causing high levels of nutritional stress, and without the need for complex designs that target or avoid particular groups of cattle.

The robustness of the antibody response in pregnant and lactating females (Fig. 3d) is particularly promising, given that hormonal changes and lactational stresses can induce changes in populations of immune cells and can trigger immunosuppression in cattle [35,37]. These results are also encouraging for pastoralists, given the high value of these animals and their critical importance for food security and income for households [7]. It is also pregnant and lactating females which often have the highest risk of exposure to MCF as they more typically remain at the household to provide food for the family and, consequently, are less likely to be moved away from the household to avoid wildebeest [7,26]. This valuable finding indicates that even the most immunologically vulnerable and economically important animals exhibit a comparable response to the primary vaccination as other animals. This suggests that the vaccine could help mitigate some of the most significant impacts of MCF, particularly those affecting milk availability and production.

Further reassurance of the potential utility of the vaccine is provided by the very few adverse effects being reported even though cattle were vaccinated at a time of high nutritional stress and the study involved vaccination of vulnerable and valuable animals, such as pregnant and lactating females, which were not included in previous field trials [23,24]. No acute reactions were observed immediately after vaccine administration and no incidents of abortion were reported in any of the 300 pregnant animals that were vaccinated (Supplementary Information, Table 3). These findings are especially relevant for farmers, as abortion not only results in the loss of valuable livestock and productivity but could also hinder farmer adoption of the vaccine [4]. The lack of adverse effects reported therefore reaffirms vaccine safety for use in local Tanzanian cattle, as explicitly demonstrated in previous studies, [23,24]. Further discussion of results of the clinical and diagnostic investigations is provided in the Supplementary Information (Section 6.4).

The second element of the study (P2) allowed us to explore, for the first time, two further issues of relevance for the real-world implementation and design of MCF vaccination programs, generating data on: (i) the duration of the antibody response under field settings and (ii) antibody responses elicited by booster vaccination regimens. Drawing on results from experimental challenge trials [25], our expectation was that, by 6–12 months after vaccination, antibody levels would have declined to levels well below peak post-vaccination responses. However, although a decline at 11 months post-primary vaccination was detected (i.e. s/*p* values falling by 18 % from a mean of 0.60 at T2 to 0.49 at T3 - Comparison 2), these levels remained significantly higher than baseline levels (Fig. 5). This decline was also less marked than that observed in challenge trials carried out in Holstein-Friesian cattle where average antibody titres had fallen by more than 75 % (from a peak of >1/400 to less than 1/100) by 120 days post-vaccination and with seven out of eight cattle tested at 252 days post- vaccination having no detectable AlHV-1 specific antibodies [25]. The sustained antibody levels observed in this study after primary vaccination, therefore offer valuable insights into the duration of immunity in real-world conditions, a critical aspect that had not yet been thoroughly investigated outside of controlled settings [25].

The second key result from P2 was the finding that a single-dose boost vaccination resulted in substantial increases in antibody levels, with mean s/*p* values at T4 (0.80) greater than the mean peak values (0.61) recorded after the primary course (T2). Furthermore, there was only a small increase in overall mean s/p values observed after the second booster dose at T5 (0.88) compared to the single booster at T4 (Fig. 4b). These results suggest that, in contrast to the primary course where a two-dose regimen is essential for eliciting an immune response [22,25], a single dose boost vaccination is likely to be sufficient to maintain high levels of immunity in African short-horn zebu cattle. Several mechanisms likely contributed to the limited immune response of cattle to the second dose boost vaccination at T5. Among these, with the immune system already stimulated by the primary course of vaccination, the first booster dose after 12 months likely served to restimulate the memory response, rapidly inducing high antibody levels, while the second booster dose had a minimal effect. Moreover, high levels of natural exposure to MCF, as observed in this study, may play a critical role by providing a natural boost effect that helps to maintain antibody levels between vaccination doses [24,29]. Further investigation is warranted to explore the role of natural exposure in maintaining post-vaccination responses.

Although MCF is often considered a fatal disease of cattle, this study does provide further evidence for non-fatal AlHV-1 infections, with seropositivity recorded in 34.6 % of cattle at baseline (T1) and qPCR confirmation of one non-fatal case of AlHV-1 infection (A-250). In this study, baseline seropositivity was higher than recorded previously in the Tarangire ecosystem [24] and may be explained by greater contact and exposure of cattle with wildebeest calves in mixed grazing areas of the Serengeti ecosystem, particularly around the village of Ololosokwan (Fig. 1). However, mean s/*p* values at baseline were low and we should be cautious about estimates of seroprevalence based on the >0.06 s/p threshold for seropositivity set using MCF negative cattle in the UK [31]. Nonetheless, s/p values for some individuals were comparable with those seen in vaccinated animals, suggesting that non-fatal exposure does occur which could have potential implications for vaccination strategies [24].

Overall, the findings from the second (P2) element of the study suggest that the duration of immunity provided by a primary course of vaccination under field settings, for all categories of cattle, is likely to span the whole MCF risk period during the annual wildebeest calving season [24,38] with annual single–dose boost vaccinations likely to be sufficient to maintain immunity across subsequent years. This prolonged protection could also be advantageous for communities that live in areas where the MCF risk is less seasonal, for example areas with resident wildebeest populations year-round [39], or in game farming/ranching situations where contact between cattle and wildebeest may not have a seasonal basis [6,38]. The extended duration of immunity observed could also offer greater flexibility and affordability in scheduling vaccinations, allowing cattle to be vaccinated further in advance of the risk period at times when they are more accessible and in better condition. This flexibility could also allow options for simultaneous delivery of the MCF vaccine with other vaccination or animal health delivery programs. This integration would streamline the process for farmers and animal health service providers and would likely improve uptake and the cost-effectiveness of delivery [4].

Adoption of MCF vaccination has the potential to provide substantial benefits to pastoralists living in high-risk areas, supporting the health and productivity of cattle on which the lives, food security and wellbeing of pastoralists depend, particularly at a time when the survival of pastoral systems is being threatened by escalating droughts and dwindling pasture access [15,16]. The vaccine would allow cattle to graze in more productive pastures during the MCF risk season and regain condition at critical times of year, with likely improvements in conception rates and milk production. The advent of the vaccine could reduce the need for cattle to be moved away from the main household during the 3–5 months of the MCF risk season, providing a more reliable year-round source of high-quality food and protein for children.

In summary, our findings demonstrate high levels of immunogenicity and safety of the AlHV-1 vaccine against MCF in locally owned cattle in Tanzania, as supported by the minimal adverse reactions and substantial increases in immune response post-primary vaccination course. Our findings reveal that this significant immune response is consistent across several individual host factors and immunologically vulnerable groups. The study further indicates a more prolonged duration of the immune response than previously observed and that an annual single-dose booster vaccination should be sufficient to sustain immunity in subsequent years.

## Ethics statement

The study was approved by the University of Glasgow College of Medical, Veterinary and Life Sciences ethics committee (200200144) and the School of Biodiversity, One Health, and Veterinary Medicine animal ethics committee (EA0121). In Tanzania, the study was conducted with approval from the Kibing'oto Nelson Mandela and Ceda Health Research Ethical Committee through the Nelson Mandela African Institution of Science and Technology (KNCHREC42/03/2021), the Tanzania Wildlife Research Institute and the Commission for Science and Technology (COSTECH 2021–027-NA- 2015-043). Approval was obtained through the Tanzania Medicines and Medical Devices Authority for importation and research use of the MCF vaccine (TMDA- WEB0021/D/SIPER/1144) and for importation of reagents for MCF serological and PCR analyses (TMDA-WEW0023/MDR/SIPER/4720). All animal research was carried out in line with the ARRIVE guidelines and in accordance with the U.K. Animals (Scientific Procedures) Act, 1986.

## CRediT authorship contribution statement

**Samuel Bainbridge:** Writing – review & editing, Writing – original draft, Visualization, Project administration, Methodology, Investigation, Formal analysis, Data curation, Conceptualization. **Tauta Mappi:** Writing – review & editing, Project administration, Methodology, Investigation, Conceptualization. **Sarah Cleaveland:** Writing – review & editing, Writing – original draft, Supervision, Project administration, Methodology, Investigation, Funding acquisition, Formal analysis, Data curation, Conceptualization. **Choby Chubwa:** Writing – review & editing, Project administration, Conceptualization. **Alicia Davis:** Writing – review & editing, Project administration, Methodology, Investigation, Funding acquisition, Conceptualization. **Dawn Grant:** Writing – review & editing, Methodology, Investigation, Data curation. **Tito Kibona:** Writing – review & editing, Project administration, Investigation. **Shedrack Bwatota:** Writing – review & editing, Project administration, Investigation. **Freja Larsen:** Writing – review & editing, Investigation. **Samson Lyimo:** Writing – review & editing, Project administration, Methodology, Investigation, Data curation. **Fadhili Mshana:** Writing – review & editing, Project administration, Investigation. **Ann Percival:** Writing – review & editing, Methodology, Investigation, Data curation. **Gabriel Shirima:** Writing – review & editing, Writing – original draft, Project administration, Methodology, Funding acquisition, Formal analysis, Data curation, Conceptualization. **Bakari Mtili:** Writing – review & editing, Project administration, Investigation. **Felix Jackson Musyangi:** Writing – review & editing, Investigation. **Rigobert Tarimo:** Writing – review & editing, Project administration, Investigation. **Felix Lankester:** Writing – review & editing, Writing – original draft, Project administration, Investigation, Funding acquisition, Formal analysis, Conceptualization. **George Russell:** Writing – review & editing, Writing – original draft, Resources, Project administration, Methodology, Funding acquisition, Formal analysis, Data curation, Conceptualization.

## Declaration of competing interest

The authors declare the following financial interests/personal relationships which may be considered as potential competing interests: Sarah Cleaveland reports financial support was provided by UK Research and Innovation. Sarah Cleaveland reports financial support was provided by Wellcome Trust. Sarah Cleaveland reports financial support was provided by Biotechnology and Biological Sciences Research Council. Sarah Cleaveland reports financial support was provided by Global Challenges Research Fund. If there are other authors, they declare that they have no known competing financial interests or personal relationships that could have appeared to influence the work reported in this paper.

## Data Availability

Data will be made available on request.

## Appendix A Supplementary data

Supplementary material: Detailed model descriptions, results tables, clinical events, additional analyses, demographic data, and consent forms supporting the findings of this study. Image 1

## References

[1] Russell G.C., Stewart J.P., Haig D.M. (2009). Malignant catarrhal fever: a review. The Veterinary Journal.

[2] Gong M. (2023). Wildebeest-derived malignant catarrhal fever: a bovine peripheral T cell lymphoma caused by cross-species transmission of Alcelaphine Gammaherpesvirus 1. Viruses.

[3] Gong M. (2024). Unraveling clonal CD8 T cell expansion and identification of essential factors in γ-herpesvirus-induced lymphomagenesis. Proc Natl Acad Sci U S A.

[4] Decker C. (2021). Predicting uptake of a malignant catarrhal fever vaccine by pastoralists in northern Tanzania: opportunities for improving livelihoods and ecosystem health. Ecol Econ.

[5] Bedelian C., Nkedianye D., Herrero M. (2007). Maasai perception of the impact and incidence of malignant catarrhal fever (MCF) in southern Kenya. Prev Vet Med.

[6] Cleaveland S., Kusiluka L., Kuwai J.O., Bell C., Kazwala R. (2001).

[7] Lankester F. (2015). The economic impact of malignant catarrhal fever on pastoralist livelihoods. PloS One.

[8] Chege P.M., Kimiywe J.O., Ndungu Z.W. (2015). Influence of culture on dietary practices of children under five years among Maasai pastoralists in Kajiado, Kenya. Int J Behav Nutr Phys Act.

[9] Galiè A., Farnworth C.R., Njiru N., Alonso S. (2021). Intra-household handling and consumption dynamics of Milk in Peri-urban informal Markets in Tanzania and Kenya: a gender Lens. Sustainability.

[10] Lawson D.W. (2014). Ethnicity and child health in northern Tanzania: Maasai pastoralists are disadvantaged compared to Neighbouring ethnic groups. PloS One.

[11] Cassidy E. (2022).

[12] Toreti A. (2022). Drought in East Africa in.

[13] de Glanville W.A. (2020). Classification and characterisation of livestock production systems in northern Tanzania. PloS One.

[14] Veldhuis M.P. (2019). Cross-boundary human impacts compromise the Serengeti-Mara ecosystem. Science.

[15] Lankester F., Davis A. (2016). Pastoralism and wildlife: historical and current perspectives in the east African rangelands of Kenya and Tanzania. Revue Scientifique et Technique de l’OIE.

[16] Weldemichel T.G. (2020). Othering pastoralists, state violence, and the remaking of boundaries in Tanzania’s militarised wildlife conservation sector. Antipode.

[17] Holdo R.M., Holt R.D., Fryxell J.M. (2009). Opposing rainfall and plant nutritional gradients best explain the wildebeest migration in the Serengeti. Am Nat.

[18] Herrik A.L., Mogensen N., Svenning J., Buitenwerf R. (2023). Rotational grazing with cattle-free zones supports the coexistence of cattle and wild herbivores in African rangelands. J Appl Ecol.

[19] Kiffner C. (2020). Long-term persistence of wildlife populations in a pastoral area. Ecol Evol.

[20] Maitima J.M. (2009). The linkages between land use change, land degradation and biodiversity across East Africa. Afr J Environ Sci Tech.

[21] Plowright W., Herniman K.A., Jessett D.M., Kalunda M., Rampton C.S. (1975). Immunisation of cattle against the herpesvirus of malignant catarrhal fever: failure of inactivated culture vaccines with adjuvant. Res Vet Sci.

[22] Haig D.M. (2008). An immunisation strategy for the protection of cattle against alcelaphine herpesvirus-1-induced malignant catarrhal fever. Vaccine.

[23] Cook E. (2019). A randomised vaccine field trial in Kenya demonstrates protection against wildebeest-associated malignant catarrhal fever in cattle. Vaccine.

[24] Lankester F. (2016). A field vaccine trial in Tanzania demonstrates partial protection against malignant catarrhal fever in cattle. Vaccine.

[25] Russell G.C. (2012). Duration of protective immunity and antibody responses in cattle immunised against alcelaphine herpesvirus-1-induced malignant catarrhal fever. Vet Res.

[26] Haseeb A. (2019). Economic burden of livestock disease and drought in northern Tanzania. J Dev Agric Econ.

[27] Dyce K.M., Sack W.O., Wensing G. (2009).

[28] Eversole D.E., Browne M.F., Hall J.B., Dietz R.E. (2000). Body condition scoring beef cows. https://www.thecattlesite.com/articles/674/body-condition-scoring-beef-cows/.

[29] Lankester F. (2016). The efficacy of alcelaphine herpesvirus-1 (AlHV-1) immunization with the adjuvants Emulsigen ® and the monomeric TLR5 ligand FliC in zebu cattle against AlHV-1 malignant catarrhal fever induced by experimental virus challenge. Vet Microbiol.

[30] Traul D.L. (2005). A real-time PCR assay for measuring alcelaphine herpesvirus-1 DNA. J Virol Methods.

[31] Russell G.C., Percival A., Grant D.M. (2025). Indirect ELISA for analysis of malignant catarrhal fever virus-specific antibodies in a range of species. J Virol Methods.

[32] Russell G.C. (2021). Analysis of immune responses to attenuated alcelaphine herpesvirus 1 formulated with and without adjuvant. Vaccine X.

[33] R Core Team (2022).

[34] Bates D., Mächler M., Bolker B., Walker S. (2015). Fitting linear mixed-effects models using lme4. J Stat Softw.

[35] Vlasova A.N., Saif L.J. (2021). Bovine immunology: implications for dairy cattle. Front Immunol.

[36] Carroll J.A., Forsberg N.E. (2007). Influence of stress and nutrition on cattle immunity. Vet Clin North Am Food Anim Pract.

[37] Roth J.A. (1999).

[38] Wambua L., Wambua P.N., Ramogo A.M., Mijele D., Otiende M.Y. (2016). Wildebeest-associated malignant catarrhal fever: perspectives for integrated control of a lymphoproliferative disease of cattle in sub-Saharan Africa. Arch Virol.

[39] Hopcraft J.G.C. (2014). Competition, predation, and migration: individual choice patterns of Serengeti migrants captured by hierarchical models. Ecological monographs.

