# Supplementary material for: Field vaccination of locally-owned cattle against malignant catarrhal fever under environmentally challenging conditions in Tanzania
Source: Vaccine. 2025 Jan 25;45:None. doi: 10.1016/j.vaccine.2024.126587 (PMC11773376; doi:10.1016/j.vaccine.2024.126587)
Supplement: Supplementary file 1 — Supplementary material: Detailed model descriptions, results tables, clinical events, additional analyses, demographic data, and consent forms supporting the findings of this study. [file mmc1.docx]

1. **Supplementary Material**
   1. **Description and summary of models fitted to Comparisons 1-4**

**Table 1:** Description and summary of the statistical analyses used for Comparisons 1-4 investigating the effect of individual animal characteristics on the magnitude of the antibody response following a primary two-dose vaccination, single-dose boost vaccination and two-dose boost vaccination of the Malignant Catarrhal Fever vaccine in local cattle in Northern Tanzania.

| **Model** | **Model Formula** | **Description** |
| --- | --- | --- |
| **Comparison 1** | | |
| Model 1 | s/p difference~ Age + Village + Body condition + Breed + Reproductive Status  +(1\|Household ID) | This is a timepoint difference log-normal model, with the difference between T1-T2 log-transformed fitted as the response variable. Household ID was used as a random effect.  During residual diagnostics, significant deviations from normality were detected, indicated by a significant KS test and quantile deviations in the residual vs. predicted plot. These findings suggest that the model’s residuals do not satisfy the assumption of normality, which is a critical assumption for linear mixed-effects models.  To address these issues, we applied a log-normal transformation to the response variable which resolved this issue.  As a result of this transformation however, the one individual that did not experience an increase in antibody levels was removed from the analysis as the log-normal transformation can only take positive values. |
| Model 2 | Pre-exposed ~ Village | A binomial generalized linear model (GLM) to investigate pre- vaccination seropositivity across villages. The response variable is either pre-exposed (s/p value >0.06) (1) or not (0). |
| **Comparison 2** | | |
| Model 1 | T2 s/p value and T3 s/p value | A paired t-test was fitted after checking the data met the assumptions for the test. |
| **Comparison 3** | | |
| Model 1 | T3 s/p value and T4 s/p value | A paired t-test was fitted after checking the data met the assumptions for the test. |
| **Comparison 4** | | |
| Model 1 | s/p value ~ timepoint+(1\|Individual ID) | This model estimates the impact of timepoints T3, T4, and T5 on s/p values, which are fitted as the response variable. Individual ID is included as a random effect.  Several outliers, identified by Cook's distance, were removed due to deviations observed in residual versus predicted plots. This removal stabilized the model and resolved issues with residuals.  Only the timepoint was included in the Comparison 4 model because this comparison in the study aimed to assess whether a single booster was as effective as a two- dose booster across T3, T4, and T5. This approach focused on determining differences without unnecessary complexity.  Additionally, the smaller sample size in the Comparison 4 cohort could lead to sparse data points per variable combination, increasing the risk of overfitting |

**6.2 Consent form for Households**


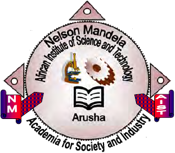

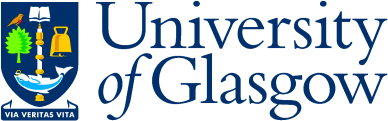


**Farmer Adoption of MCF vaccination**

**Cattle Owner Information Sheet and Consent Form - Vaccination**

**INTRODUCTION**

A few months ago, you participated in our study of malignant catarrhal fever, which is a project involving researchers from the Nelson Mandela African Institution of Science and Technology and the University of Glasgow in the UK. We are now visiting you to provide some further information about MCF vaccination and to explain the next phase of this research project.

The study is being funded through the UKRI Collective Fund and the University of Glasgow. The Tanzania Wildlife Research Institute, the Kibing’oto Nelson Mandela and Ceda Health Research Ethical Committee (KNCHREC) and the University of Glasgow, UK, have reviewed this study.

**WHY HAVE I BEEN CHOSEN?**

You are being asked to take part in this research study because you previously participated in the household survey on MCF. It is very important that you understand what is involved in the next phase of the study so please read this information sheet or have it read out to you. We encourage you to ask the project or community representative to explain words or information that you do not understand. Take as much time as you need to make a decision about whether you would like to be involved.

**WHAT WILL BE INVOLVED IN THE STUDY?**

If you agree to be in this phase of the study, you will be asked whether you would like to vaccinate your cattle against MCF. The vaccine is a new vaccine that has not been registered for commercial use in Tanzania but is currently available for limited use in Tanzania with approval of the Tanzanian Medicines and Medical Devices Directorate. We anticipate that the vaccine is likely to become available within the next month, and that we will need to charge TSh 22,000 per cattle vaccinated to cover costs. If demand is very high, we may have to limit the number of doses available to each herd.

This vaccine has been tested previously in small-scale studies in Tanzania and Kenya and has shown to be safe to use and to provide some protection from MCF transmission from wildebeest. In studies in Kenya, the vaccine showed an efficacy of 80%. To explain this a bit further, if you were to graze 100 unvaccinated cattle close to wildebeest calves, we would expect that 20 animals would die of MCF. If you were to graze 100 vaccinated animals in the same high-risk areas, we would expect that 4 would die of MCF. As this vaccine will not protect every animal, you will need to take time to decide where and how you think it might be useful for management of your herd. For example, you may decide the risk is still too high and vaccination is not worthwhile. Or you may decide to vaccinate only animals that you cannot move away from high-risk areas. Or you may choose to vaccinate animals and graze them in different areas that may be at higher risk of MCF transmission. It is your decision entirely as to which animals you choose to vaccinate and our project coordinator will be available to discuss things further with you if this is helpful. You should be aware that vaccination at this time is only likely to protect your animals during the upcoming MCF season (2022) and not in subsequent years.

We anticipate that the vaccine will be available within a few weeks and we will provide further information about the date and arrangements for the vaccination. For today, we would like to ask only how many animals you would like to vaccinate.

At the time of vaccination, you will be asked if you are willing to participate in a post-vaccination household survey that will include the same questions as those you answered earlier this year in relation to cases of MCF, livestock movements and family milk consumption. This will allow us to see whether and how vaccination of cattle has led to any changes in the way people are managing their cattle and the availability of milk. If you choose not to vaccinate any animals, we would still like to ask you to participate in this household survey.

We would like to ask a small number of participants some further in-depth questions in a conversational interview. If you are selected to participate, we may ask you if the conversation can be audio-recorded.

The vaccination involves an intramuscular dose of vaccine given at two time points, four weeks apart. Your cattle will only be protected after the second dose. We may also ask to collect a blood sample from up to ten vaccinated cattle before and after vaccination. This will allow us to check the response of cattle to the vaccine if needed. An eartag will be fitted to each vaccinated animal.

**HOW WILL THIS INFORMATION BE USED?**

The information collected today will be used to indicate the level of demand in your community for a MCF vaccine. All information relating to your vaccination choices will be kept confidential. We will keep the records under a code number rather than your name. We will keep the records in a safe place and only study staff will be allowed to look at them. Your name, or other facts that might point to you will not appear when we present this study or publish the results. The data that we collect will be stored at the University of Glasgow in Scotland (UK).

We will ask for your phone number for communication and planning purposes only. These numbers will be kept confidential and will be deleted at the end of the study.

At the end of the research study, the anonymized results will be shared with local communities and animal health leaders of the region and the country. The results of the study will also be made available in reports to the Ministry of Livestock and Fisheries and the Tanzanian Commission for Science and Technology.

**WHAT ARE THE POSSIBLE DISADVANTAGES AND RISKS OF TAKING PART IN THIS STUDY?**

There are both risks and benefits associated with this study and it is very important that you understand these.

Although the vaccine has been tested previously in cattle in Tanzania and Kenya and no serious adverse effects have been reported, any vaccine can cause adverse reactions. In previous studies, a few vaccinated cattle have shown signs of inappetence, depression, facial swelling, salivation, laboured breathing and lying down. All animals fully recovered within 24 hours. The vaccine has not been tested in pregnant cattle. We will have drugs that can treat animals showing these reactions at the time of vaccination, but it is still possible that problems may arise following vaccination which we are not able to treat.

Although the vaccine provides some protection against MCF, you must be aware that this protection is not guaranteed for all animals – it is not 100%. If vaccinated animals come into contact with wildebeest calves a small number may still die from MCF. Therefore you need to consider the risks very carefully if you choose to change the way you graze and manage your cattle.

**ARE THERE BENEFITS TO TAKING PART IN THE STUDY?**

The benefits to taking part in the study are that you will have the opportunity to vaccinate your cattle against MCF, which should reduce the risk of MCF deaths in animals that come into contact with wildebeest calves. The opportunity of vaccination may also allow you to change the way you manage and graze your cattle to access more productive grazing land and to reduce the numbers of cattle that have to be moved away from the permanent household during the MCF season. This may then allow more milk to become available for family members living at the permanent household, particularly women and children.

You will not receive any other direct benefit from participating but we hope that the information gained from this study will help new cattle vaccines against MCF becoming available to people in your communities more quickly.

**WHAT DO I DO IF I DECIDE I NO LONGER WANT TO BE INVOLVED?**

You can withdraw from the study or any part of the study at any time and you do not need to give a reason. Withdrawal will not incur any sort of penalty. If you decide to withdraw whilst project staff are still at your household, please talk to the study coordinator. If you decide to withdraw, no new information will be collected about you, and you can also ask us not to use any information we have already collected. If you would like to withdraw after we have left your household, please call Dr. Gabriel Shirima on +255- xxxxxxx. Alternatively, you can write to Dr. Gabriel Shirima, NM-AIST, PO Box 447, Arusha, Tanzania.

**WHOM DO I CALL IF I HAVE QUESTIONS OR PROBLEMS?**

For questions about your rights as a research participant, or to discuss problems, concerns or suggestions related to the research, or to obtain information or offer input about the research, contact Dr Gabriel Shirima on +255-xxxxxxx or the Kibing’oto Nelson Mandela and Ceda Health Research Ethical Committee (KNCHREC) on +255-xxxxxxx.

**On behalf of the whole project team, thank you for your time.**

**………………………………………………………………………………………………………….**

**STATEMENT OF CONSENT REGARDING PARTICIPATION IN THE STUDY**

The purpose of this study, and the study procedures, risks and benefits have been explained to me. I have been allowed to ask questions, and my questions have been answered to my satisfaction. I have been told that I may contact the Kibing’oto Nelson Mandela and Ceda Health Research Ethical Committee (KNCHREC) on +255-xxxxxxx if I have questions about my rights as a research subject, to discuss problems, concerns, or suggestions related to the research, or to obtain information or offer input about the research.

-
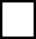
I confirm that I have read the information in this document/ that it has been read to me.
-
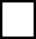
I understand that my participation is voluntary, that I am able to participate in any or none of the components of this study, and that I am free to withdraw at any time, without giving any reason and without my legal rights being affected.


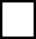


- I understand that data used for research purposes will be anonymized and no one will be able to identify me from reports and publications from the study.

| Name of subject |  | Date |  | Signature |  |
| --- | --- | --- | --- | --- | --- |
| Name of Person taking consent |  | Date |  | Signature |  |
| Witness (if applicable) |  | Date |  | Signature |  |

(1 copy for the study; 1 copy for the participant)

**STATEMENT OF INTENTION REGARDING VACCINATION OF CATTLE AGAINST MCF**

- - I confirm that I will purchase vaccine sufficient to vaccinate cattle against MCF.
  - I understand that the vaccination will involve 2 doses of vaccine given 4 weeks apart.
  - I understand that the vaccine will cost TSh 22,000 per animal vaccinated.
  - I understand that the vaccine provides partial protection of 80% against MCF and I understand what this means.
  - I understand that the vaccine will only provide protection against MCF during the coming MCF season and may not protect cattle in subsequent years.

| Name of subject |  | Date |  | Signature |  |
| --- | --- | --- | --- | --- | --- |
| Name of Person taking consent |  | Date |  | Signature |  |
| Witness (if applicable) |  | Date |  | Signature |  |

*To be completed by the research team:*

Household ID number (to be completed by research team)

Date of previous questionnaire survey(s) Date 1: / / Date 2: / /(1 copy for the study; 1 copy for the participant)

**STATEMENT FOR CONSENT TO VACCINATE CATTLE AGAINST MCF**

- I confirm that I have read the information in this document/ that it has been read to me.
- I understand that my participation is voluntary
- I understand that data used for research purposes will be anonymized and no one will be able to identify me from reports and publications from the study.
- I confirm my agreement for cattle to be vaccinated against MCF.
- I understand that the vaccination will involve 2 doses of vaccine given 4 weeks apart.
- I understand that there will be no charge to have my animals vaccinated.
- I understand that the vaccine provides partial protection of 80% against MCF and I understand what this means.
- I understand that the vaccine will only provide protection against MCF during the coming MCF season and may not protect cattle in subsequent years.

| Name of subject |  | Date |  | Signature |  |
| --- | --- | --- | --- | --- | --- |
| Name of Person taking consent |  | Date |  | Signature |  |
| Witness (if applicable)  *To be completed by the research team:* |  | Date |  | Signature |  |

Household ID number (to be completed by research team)

(1 copy for the study; 1 copy for the participant)

**STATEMENT FOR CONSENT FOR THE IN-DEPTH INTERVIEWS**

- I confirm that I have read the information in this document/ that it has been read to me.
- I understand that my participation is voluntary, that I am able to participate in any or none of the components of this study, and that I am free to withdraw at any time, without giving any reason and without my legal rights being affected.
- I understand that data used for research purposes will be anonymized and no one will be able to identify me from reports and publications from the study.
- I confirm that I am willing to participate in an in-depth interview.
- I agree that the conversation can be audio-recorded.

| Name of subject |  | Date |  | Signature |  |
| --- | --- | --- | --- | --- | --- |
| Name of Person taking consent |  | Date |  | Signature |  |
| Witness (if applicable)  *To be completed by the research team:* |  | Date |  | Signature |  |

Household ID number (to be completed by research team)

1. copy for the study; 1 copy for the participant

**6.3 Clinical Events**

**A-133:**

- - - **Age:** 2 years
    - **Village:** Arash
    - **Vaccinated:** 29/12/2021 (First dose)
    - **Sex:** Male
    - **Breed:** Indigenous breed

**Summary:** An adverse reaction was observed by the farmer on 30/12/2021 after the first primary vaccination prime dose. Observed clinical signs included loss of appetite. The farmer administered OTC 5%, Penicillin-Streptomycin and Tylosine for three days consecutively. From a follow up by the team on 24/01/2022 no obvious clinical signs were observed. Cardinal parameters indicated a temperature of 38.9°C and a respiration rate of 24 cycles/min.

**A-18:**

- - - **Age:** 3 years
    - **Village:** Arash
    - **Vaccinated:** 28/12/2021 (First dose)
    - **Sex:** Male
    - **Breed:** Indigenous breed

**Summary:** An adverse reaction was observed by the farmer on 29/12/2021 after the first primary vaccination prime dose. The clinical signs observed included a loss of appetite and dizziness, with diarrhoea observed on 01/01/2022. No drugs were prescribed to the animal. From a follow up by the team on 24/01/2022 no obvious clinical signs were observed, and the animal was grazing well. Cardinal parameters indicated a temperature of 38.3°C, respiration rate of 28 cycles/min and a pulse rate of 60 bpm. Further follow up by the team on 26/01/2022 indicated the animal was doing well and on 05/02/2022 no obvious clinical signs were observed; therefore, a second dose of vaccine was administered.

**A-29:**

- - - **Age:** 9 months
    - **Village:** Arash
    - **Vaccinated:** 28/12/2021 (First dose)
    - **Sex:** Female
    - **Reproductive Status:** Not lactating
    - **Breed:** Indigenous breed

**Summary:** An adverse reaction was observed on 29/12/2021 after the first primary vaccination prime dose. The clinical signs observed included bilateral lachrymation, bilateral corneal opacity, loss of menace on the right eye (reported by the farmer and seen by a veterinarian after a phone call on 29/12/2021) and a snoring sound in the trachea when breathing out (auscultation using a stethoscope was carried out on 24/01/2022). A suspected MCF case form was filled out and a thorough follow-up was arranged by the field team. From the follow up, opacity was spreading from the middle to the peripheral, treatment was given through eye washing with clean water and topical application of 2.5% OTC powder which was applied by the farmer daily. Cardinal parameters taken indicated a temperature of 37.8°C and a respiration rate of 18 cycles/min. On a second follow up by the team on 26/01/2022, the animal showed reduced lachrymation and opacity, the menace response had gained, and serum, plasma and buffy coat samples were taken. Cardinal parameters indicated a temperature of 38.9°C and a respiration rate of 24 cycles/min. On a further follow up on 05/02/2022 there was no lachrymation observed, a reduced opacity > 80%, therefore a second dose of vaccine was given.

**O-1157:**

- - - **Age:** 4 years
    - **Village:** Ololosokwan
    - **Vaccinated:** 19/01/2022 (First dose)
    - **Sex:** Female
    - **Breed:** Indigenous breed

**Summary:** An adverse reaction was observed on 25/01/2022 after the first primary vaccination prime dose. The clinical signs observed included swelling around both sides of the neck region, around the head, which progressed to the dewlap, front leg, and hind legs. The treatment administered by the farmer was OTC 5%. From the follow up of the team on 23/02/2022, the animal’s pre-scapular lymph nodes were swollen, but despite other swellings remaining they were significantly reduced, and a second dose of vaccination was administered. On a follow-up phone call with the farmer on 24/02/2022 it was reported that the animal was doing well, and no further reaction was seen.

**P-602:**

- **Age:** 8 years
- **Village:** Piyaya
- **Vaccinated:** 12/12/2022 (Second dose)
- **Sex:** Female
- **Breed:** Indigenous breed

**Summary:** An adverse reaction was observed 13/02/2022 after the second primary vaccination boost dose. The clinical signs observed included loss of appetite and a rough hair coat. No treatment was given and from the follow up of team on 15.02.2022 the animal was grazing, and the hair coat was smooth. Cardinal parameters indicated a temperature of 38.4°C and a respiration rate was unable to be recorded due to difficulty in handling the animal.

**A-352:**

- - - **Age:** 9 years
    - **Village:** Arash
    - **Vaccinated:** 03/01/2022 (First dose)
    - **Sex:** Female
    - **Breed:** Indigenous breed

**Summary:** An adverse reaction was noted for this animal after the first dose of vaccination, but no clinical record was filled out and the animal was given a second dose of vaccination on 10/02/2022.

**6.4 Death Reports**

**A-255:**

- - - **Age:** 2 years
    - **Village:** Arash
    - **Vaccinated:** 29/12/2021 (First dose)
    - **Sex:** Female
    - **Breed:** Indigenous breed

**Summary:** The farmer reported that the animal did not show any clinical signs, and it was found lying down (lateral recumbency). The animal was slaughtered, and its internal organs showed no lesions. No samples were taken.

**O-1022:**

- - - **Age:** 5 years
    - **Village:** Ololosokwan
    - **Vaccinated:** 15/01/2022 (First dose)
    - **Sex:** Female
    - **Breed:** Indigenous breed

**Summary:** The farmer reported that the animal had grazed well in rush pasture but had a distended abdomen (bloat), then died on 25/01/2022. The post-mortem investigation conducted on 25/01/2022 indicated that the animal had diarrhoea (faecal matter was found near the animal), the skin was smooth, and no lesions were observed near the eye or mouth. The animal’s rumen was distended, the abomasal contents were dried, no lesions were seen on the lungs, liver, spleen, kidney, or lymph nodes. Samples were taken of the pre-scapular lymph node, liver, lungs, kidney, and spleen.

**0-1091:**

- - - **Age:** 6 years
    - **Village:** Ololosokwan
    - **Vaccinated:** 19/01/2021 (First dose)
    - **Sex:** Female
    - **Breed:** Indigenous breed

**Summary:** The farmer reported on 27/01/2022 that the animal was suffering from bilateral lachrymation, corneal opacity (orientation not specified), and a loss of appetite. On 02/02/2022 there was a loss of sight in both eyes, blood, diarrhoea, and death on 03/02/2022. No samples were taken as the team was not informed and animals were too dispersed from the village to reach.

**0-999:**

- **Age:** 1 years
- **Village:** Ololosokwan
- **Vaccinated:** 15/01/2021 (First dose)
- **Sex:** Female
- **Breed:** Indigenous breed

**Summary:** The farmer reported no clinical signs, and the animal died on 19/01/2022. The carcass was opened by residents who ate the meat. No lesions were reported on the animal’s internal organs. No sample was able to be taken.

**0-986:**

- - - **Age:** 10 months
    - **Village:** Ololosokwan
    - **Vaccinated:** 15/01/2022 (First dose)
    - **Sex:** Male
    - **Breed:** Indigenous breed

**Summary:** The farmer reported that the animal grazed in rush pasture, it developed bloat and before death showed neck extension. The animal died on 20/01/2022. Upon opening the carcass, the lungs were enlarged (increased interstitial space). The carcass was consumed by residents and no report was submitted to the field team before the second vaccination therefore no sample was taken.

**Interpretation of Clinical Events:**

Five clinical incidents reported by farmers in the interval between the first and second doses of the primary vaccination (5/1634, 0.31% of vaccinated cattle) could be attributed to possible vaccine reactions. These were mild and non- specific, consistent with clinical events reported following use of this vaccine in previous studies [24,25,30] and potentially induced by the Emulsigen® adjuvant as suggested by Cook et al. (2019) [24]. Of the five fatalities investigated between the first and second doses of the primary course, two were associated with bloating following a surge in pasture growth after the rains. One fatality, a case of suspected MCF (O-1091) which showed clinical signs 8 days after the first vaccination dose, is thought to have resulted from early exposure to wildebeest calves at a time when the animal would not yet have been protected from vaccine-induced antibody. Although in our study, the consenting process and community meetings provided information to owners that cattle would be protected against MCF only two weeks after the second dose, this underscores the importance of clear communication of timelines around protection, and completion of the primary course of vaccination in a timely manner to reduce vulnerability of cattle between doses. The other two reported fatalities could not be investigated, although the owners gave no indication that they associated these deaths with vaccination.

While a full analysis of post-vaccination mortality was not possible in this study, the small number of clinical events reported by owners provides reassurance of vaccine safety. This is further supported by an absence of any reports of possible adverse effects following the boost vaccination in P2. However, some caveats need to be considered in relation to the lack of volunteered information from farmers in P2, as the growing confidence of pastoralists in the safety of the vaccine may have led to a perception that it was not worthwhile reporting mild events or that these events were not associated with vaccination. Nonetheless, we draw confidence from the finding that when, during the administration of the second dose boost vaccination (**T4**), the question was specifically asked (for each of the 271 animals) as to occurrence of any illness since vaccination, no incidents were reported.

- 1. **Difference in AlHV-1 Pre-exposure By Village**

This section outlines the binomial GLM applied to the Comparison 1 cohort (N=390) to investigate whether there was a statistically significant difference between the number of animals that were considered pre-exposed to AlHV-1 (s/p > 0.06) and their village of origin. The response variable was either pre-exposed (1) or not (0), and village of origin was used as the sole explanatory variable.

The analysis showed that there was a statistically significant difference between the reference level village (Arash) and Ololosokwan. Cattle from Ololosokwan were significantly more likely to be classified as pre-exposed compared to Arash (Estimate = 0.66, Std. Error =0.32, z = 2.054, p = 0.039 *). Conversely, there was no statistical difference between the number of cattle classified as pre-exposed in Piyaya compared to Arash (Estimate = -0.075, Std. Error=0.24, z =-0.31, p = 0.75).

**Table 2**: The table presents the outcomes of the binomial logistic regression model applied to the Comparison 1 cohort of cattle (N=390), The binary response variable indicated whether an individual was pre-exposed (1) or not (0). The model was fit using a binomial family, with a logit link function to model the log-odds of pre-exposure. It includes estimates, 95% confidence intervals, and P-values.

| **Parameter** | **Estimate** | **Std. Error** | **z-value** | **P-value** | **2.5% CI** | **97.5% CI** |
| --- | --- | --- | --- | --- | --- | --- |
| *(Intercept)* | -0.70 | 0.15 | -4.79 | 1.67e-06 *** | -0.99 | -0.48 |
| *Village- Ololosokwan* | 0.66 | 0.32 | 2.054 | 0.04 * | 0.028 | 1.29 |
| *Village - Piyaya* | -0.075 | 0.24 | -0.31 | 0.75 | -0.55 | 0.39 |

CI = Confidence Interval.

- 1. **Demographic Distribution of Vaccinated Animals (P1)**

**Table 3:** Summary of individual cattle factors and their distribution across the study sample that were fully vaccinated (prime and boost regimen) with the AlHV-1 malignant catarrhal fever vaccine for period 1 of the project (N=1634).

| **Variable** | **Count** | **Percentage of Sub-Group (%)** |
| --- | --- | --- |
| **Body Condition** | | |
| *Emaciated* | 185 | 11.32 |
| *Thin* | 459 | 28.09 |
| *Moderate* | 834 | 51.04 |
| *Good* | 151 | 9.24 |
| *Missing Data* | 5 | 0.31 |
| **Sex** | | |
| *Male* | 379 | 23.19 |
| *Female* | 1249 | 76.44 |
| *Missing Data* | 6 | 0.37 |
| **Male Reproductive Status** | | |
| *Not Castrated* | 261 | 69.05 |
| *Castrated* | 105 | 27.78 |
| *Missing Data* | 12 | 3.175 |
| **Female Reproductive Status** | | |
| *Pregnant* | 300 | 24.02 |
| *Lactating* | 350 | 28.02 |
| *Not Active* | 580 | 46.44 |
| *Missing Data* | 19 | 1.521 |
| **Village** | | |
| *Piyaya* | 347 | 21.24 |
| *Arash* | 558 | 34.15 |
| *Ololosokwan* | 729 | 44.61 |
| **Breed** | | |
| *Indigenous* | 1544 | 94.49 |
| *Exotic* | 4 | 0.25 |
| *Crossbreed* | 71 | 4.35 |
| *Borana* | 13 | 0.80 |
| *Missing Data* | 2 | 0.12 |
| **Age** | | |
| *Min.* | 0.50 | N/A |
| *1st Qu.* | 2.00 | N/A |
| *Median* | 4.00 | N/A |
| *Mean* | 4.41 | N/A |
| *3rd Qu.* | 7.00 | N/A |
| *Max.* | 15.00 | N/A |
| *NA's* | 7 | N/A |

- 1. **Linear Mixed Model Results**

**Table 4:** Summary of results from the Comparison 1 model, analysing the impact of individual animal characteristics on the magnitude of the antibody response following a two-dose AlHV-1 primary vaccination. Estimates are presented on the log-transformed scale of the s/p difference antibody response.

| **Parameter** | **Estimate** | **Std. Error** | **Degrees of freedom** | **t-value** | **P-value** | **2.5% CI** | **97.5% CI** |
| --- | --- | --- | --- | --- | --- | --- | --- |
| (Intercept) | -0.71 | 0.29 | 346.69 | -2.44 | 0.02 | -1.28 | -0.16 |
| Age (years) | 0.04 | 0.04 | 374.17 | 0.94 | 0.35 | -0.04 | 0.11 |
| Village- Ololosokwan | 0.09 | 0.12 | 33.11 | 0.77 | 0.45 | -0.13 | 0.31 |
| Village-Piyaya | -0.03 | 0.1 | 21.6 | -0.31 | 0.76 | -0.23 | 0.17 |
| Body Condition-Linear | 0.06 | 0.09 | 343.8 | 0.67 | 0.50 | -0.11 | 0.23 |
| Body Condition-Quadratic | -0.04 | 0.06 | 376.75 | -0.59 | 0.56 | -0.16 | 0.09 |
| Body Condition-Cubic | -0.06 | 0.04 | 376.83 | -1.33 | 0.18 | -0.14 | 0.03 |
| Breed-Crossbreed | -0.15 | 0.33 | 374.61 | -0.46 | 0.65 | -0.79 | 0.49 |
| Breed-Indigenous | -0.05 | 0.26 | 368.42 | -0.18 | 0.86 | -0.54 | 0.47 |
| Reproductive Status-Lactating | 0.07 | 0.13 | 366.48 | 0.55 | 0.58 | -0.18 | 0.33 |
| Reproductive Status-Not active (females) | 0.12 | 0.11 | 365.12 | 1.06 | 0.29 | -0.1 | 0.33 |
| Reproductive Status-Not castrated | 0.12 | 0.11 | 365.83 | 1.04 | 0.30 | -0.1 | 0.34 |
| Reproductive Status-Pregnant | 0.01 | 0.13 | 367.33 | 0.08 | 0.94 | -0.24 | 0.26 |
| **Random Effect-** Household ID | | | | | | | |
|  | **Variance** | **Std.**  **Deviation** |  |  |  |  |  |
| Intercept | 0.040 | 0.20 |  |  |  |  |  |
| Residual | 0.15 | 0.39 |  |  |  |  |  |

CI = Confidence Interval

**Table 5:** Summary of the results from the Comparison 4 model investigating the effect of a single-dose booster versus a second-dose boost vaccination on the antibody response of cattle.

| **Parameter** | **Estimate** | **Std. Error** | **Degrees of freedom** | **t-value** | **P-**  **value** | **2.5% CI** | **97.5% CI** |
| --- | --- | --- | --- | --- | --- | --- | --- |
| *(Intercept)* | 0.65 | 0.027 | 49.69 | 23.40 | <2e-16 *** | 0.59 | 0.70 |
| Timepoint-*T4* | 0.19 | 0. 015 | 80 | 12.14 | <2e-16 *** | 0.16 | 0.22 |
| Timepoint-*T5* | 0.23 | 0.015 | 80 | 14.96 | <2e-16 *** | 0.20 | 0.26 |
| **Random Effect** - ID | | | | | | | |
|  | **Variance** | **Std. Deviation** |  |  |  |  |  |
| Intercept | 0.026 | 0.16 |  |  |  |  |  |
| Residual | 0.0050 | 0.071 |  |  |  |  |  |

CI = Confidence Interval.
